# Supplementary material for: A Profile Hidden Markov Model to investigate the distribution and frequency of LanB-encoding lantibiotic modification genes in the human oral and gut microbiome
Source: PeerJ. 2017 Apr 27;5:e3254. doi: 10.7717/peerj.3254 (PMC5410138; doi:10.7717/peerj.3254)
Supplement: Table S3 [file peerj-05-3254-s003.docx]

|  | pHMM Hits | PF04738 Hits | BlastP Hits | pHMM Hit/Mb | PF04738 Hit/Mb | BlastP Hit/Mb |
| --- | --- | --- | --- | --- | --- | --- |
| Attached Keratinized Gingiva | 7 | 4 | 2 | 0.0244 | 0.0139 | 0.0070 |
| Buccal Mucosa | 96 | 74 | 64 | 0.0326 | 0.0251 | 0.0217 |
| Palatine Tonsils | 8 | 6 | 2 | 0.0204 | 0.0153 | 0.0051 |
| Saliva | 6 | 5 | 7 | 0.0472 | 0.0393 | 0.0550 |
| Subgingival Plaque | 33 | 22 | 9 | 0.0438 | 0.0292 | 0.0120 |
| Supragingival Plaque | 528 | 306 | 148 | 0.0309 | 0.0179 | 0.0087 |
| Throat | 5 | 2 | 0 | 0.0108 | 0.0043 | 0.0000 |
| Tongue Dorsum | 486 | 267 | 142 | 0.0276 | 0.0151 | 0.0081 |
| Stool | 399 | 288 | 231 | 0.0189 | 0.0136 | 0.0109 |
