# Supplementary figures and images for: A Profile Hidden Markov Model to investigate the distribution and frequency of LanB-encoding lantibiotic modification genes in the human oral and gut microbiome
Source: PeerJ. 2017 Apr 27;5:e3254. doi: 10.7717/peerj.3254 (PMC5410138; doi:10.7717/peerj.3254)

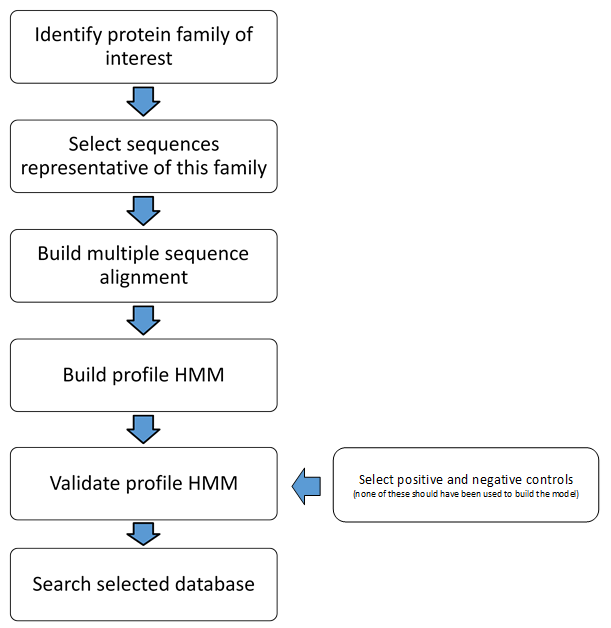

Supplement: Figure S1 [file peerj-05-3254-s007.png]

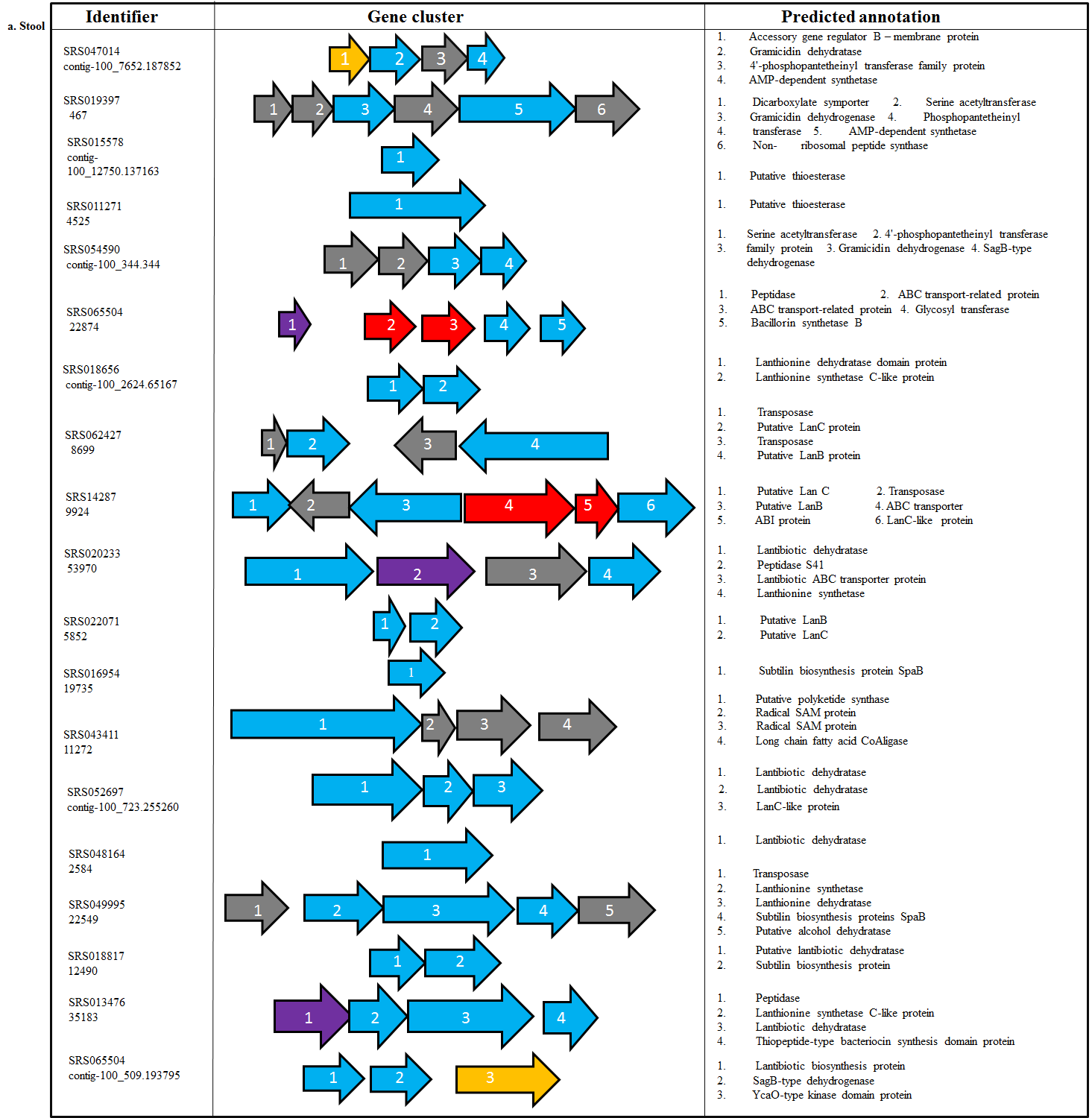

Supplement: Figure S2A — The full contig was analysed in each instance but only the area immediately surrounding the predicted LanB protein is illustrated. [file peerj-05-3254-s008.png]

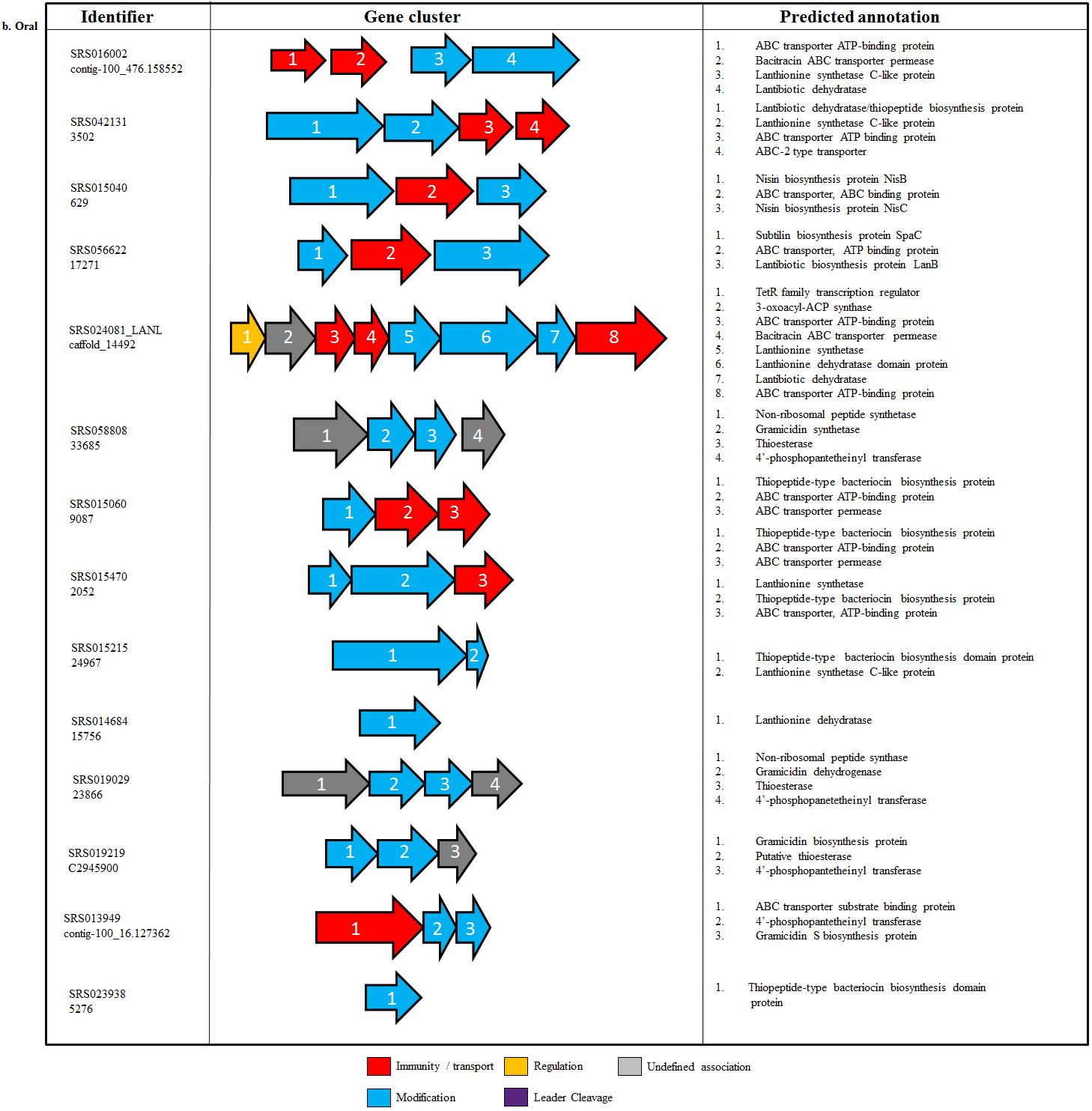

Supplement: Figure S2B — The full contig was analysed in each instance but only the area immediately surrounding the predicted LanB protein is illustrated. [file peerj-05-3254-s009.png]

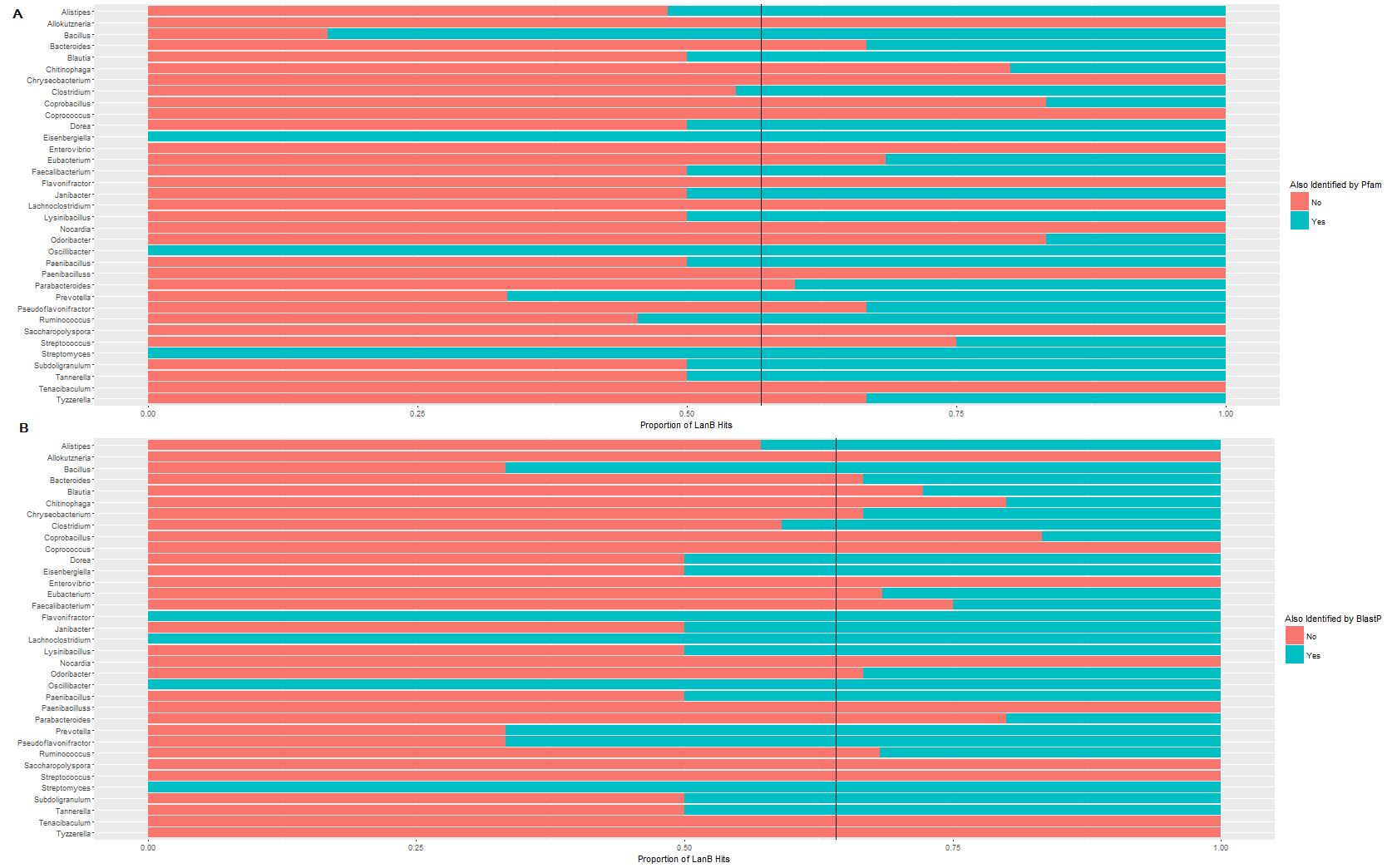

Supplement: Figure S3 — Illustrates that the proportion also identified by (A) Pfam and (B) BlastP approaches varies by producing genus. The black line shows the overall proportion of hits identified by each method. [file peerj-05-3254-s010.png]
